# Long Noncoding RNA LINC01134 Promotes Hepatocellular Carcinoma Metastasis via Activating AKT1S1 and NF-κB Signaling

**DOI:** 10.3389/fcell.2020.00429

**Published:** 2020-06-12

**Authors:** Chao Wang, Yan Chen, Kunlun Chen, Lei Zhang

**Affiliations:** ^1^Department of General Surgery, Clinical Research Center of Geriatric Diseases in Hubei Province, Tongji Hospital, Tongji Medical College, Huazhong University of Science and Technology, Wuhan, China; ^2^Department of Pediatrics, Union Hospital, Tongji Medical College, Huazhong University of Science and Technology, Wuhan, China; ^3^Department of Hepatobiliary and Pancreatic Surgery, The First Affiliated Hospital of Zhengzhou University, Zhengzhou, China; ^4^Hepatic Surgery Center, Key Laboratory of Organ Transplantation, Ministry of Education and Ministry of Public Health, Tongji Hospital, Tongji Medical College, Huazhong University of Science and Technology, Wuhan, China

**Keywords:** long noncoding RNA, hepatocellular carcinoma, metastasis, AKT1S1, NF-κB signaling

## Abstract

Hepatocellular carcinoma (HCC) is one of the most common malignancies with poor outcomes. The main causes of HCC-related deaths are recurrence and metastasis. Long noncoding RNAs (lncRNAs) are recently identified as critical regulators in cancers. However, the lncRNAs involved in HCC recurrence and metastasis are poorly understood. In this study, via analyzing The Cancer Genome Atlas Liver Hepatocellular Carcinoma dataset, we identified a novel lncRNA LINC01134, which is highly expressed in HCC tissues and correlated with microvascular invasion, macrovascular invasion, recurrence, and poor overall survival of HCC patients. Functional experiments revealed that ectopic expression of LINC01134 promotes HCC cell migration and invasion *in vitro* and HCC liver metastasis and lung metastasis *in vivo*. Knockdown of LINC01134 represses HCC cell migration and invasion *in vitro* and HCC liver metastasis and lung metastasis *in vivo*. Mechanistically, we found that LINC01134 directly binds the promoter of *AKT1S1* and activates *AKT1S1* expression. Via activating AKT1S1, LINC01134 further activates NF-κB signaling. The expression of LINC01134 is significantly positively correlated with that of AKT1S1 in HCC tissues. In line with LINC01134, AKT1S1 is also highly expressed in HCC tissues and correlated with poor survival of HCC patients. Functional rescue experiments showed that repressing AKT1S1 or NF-κB signaling abrogates the roles of LINC01134 in HCC. Taken together, these findings recognized LINC01134 as a novel oncogenic lncRNA, which indicates vascular invasion, recurrence, and poor overall survival of HCC patients. LINC01134 promotes HCC metastasis via activating AKT1S1 expression and subsequently activating NF-κB signaling. This study suggested LINC01134 as a potential prognostic biomarker and therapeutic target for HCC.

## Introduction

Liver cancer is one of the most common malignances worldwide (Bray et al., 2018). It is ranked sixth for incidence and fourth for mortality, with 841,080 estimated new cases and 781,631 estimated deaths in 2018 globally (Bray et al., 2018). Although the overall cancer mortality has fallen since 1991, the mortality for liver cancer is still increasing until now (Siegel et al., 2020). Hepatocellular carcinoma (HCC) is the major subtype and accounts for 90% of liver cancer. Very limited treatment options are available for HCC (Wen et al., 2019). Therefore, the outcomes of most HCC patients are still very poor with a 5-year survival rate of only about 18% (Siegel et al., 2020). Identification of specific molecular changes underlying HCC progression will facilitate the development of novel therapeutic strategies against HCC (Auger et al., 2015; Nasr et al., 2019).

Recent advances in human genome and transcriptome profiling have surprisingly found that most human genomes encode for noncoding RNAs (ncRNAs), but not for proteins (Iyer et al., 2015). Among these ncRNAs, long noncoding RNAs (lncRNAs) have been intensively studied in the last decade (Wang et al., 2018; Li et al., 2020). lncRNAs are a class of ncRNAs with more than 200 nucleotides (nt) in length (Ponting et al., 2009). Increasing evidences have demonstrated that lncRNAs are implicated in almost all physiological and pathological processes, including cancers (Esposito et al., 2019; Sweta et al., 2019; Zhang et al., 2019). Many lncRNAs are revealed to be dysregulated in various cancers, including HCC (Li et al., 2017; Berger et al., 2018). The lncRNAs reported to be upregulated in HCC include lncRNA-ATB, HULC, GIHCG, AWPPH, GPC3-AS1, CASC9, MCM3AP-AS1, and BZRAP1-AS1 (Yuan et al., 2014; Sui et al., 2016; Zhu et al., 2016; Zhao et al., 2017; Klingenberg et al., 2018; Xin et al., 2018; Wang W. et al., 2019; Wang Y. et al., 2019). The lncRNAs reported to be downregulated in HCC include CASC2, PSTAR, GAS8-AS1, and LINC000607 (Wang et al., 2017; Pan et al., 2018; Sun et al., 2018; Qin et al., 2019). Furthermore, many lncRNAs are reported to have critical roles in cancers, such as the regulation of cell proliferation, cell cycle, apoptosis, migration, invasion, metastasis, angiogenesis, epithelial–mesenchymal transition (EMT), autophagy, chemoresistance, and self-renewal (Cui et al., 2018; Hu et al., 2018, 2019; Kim et al., 2018; Mondal et al., 2018; Wang et al., 2018; Derderian et al., 2019; Keshavarz and Asadi, 2019). lncRNAs CASC9, PXN-AS1-L, PXN-AS1-S, RAB5IF, and MCM3AP-AS1 modulate HCC cell viability and growth via various mechanisms (Yuan et al., 2017; Klingenberg et al., 2018; Koo et al., 2019; Wang Y. et al., 2019). lncRNA-ATB, MITA1, MIR31HG, and LINC01093 were revealed to regulate HCC metastasis (Yuan et al., 2014; Yan et al., 2018; He et al., 2019; Ma et al., 2019). Thus, lncRNAs are gradually revealed to be important regulators in HCC. Although some of these lncRNAs have been studied in HCC, the total number of lncRNAs identified in human cells is more than 58,000, compared to the 21,000 protein-coding genes in human cells (Iyer et al., 2015). Therefore, other lncRNAs may also be linked to the initiation and progression of HCC.

Metastasis and recurrence are the main causes of HCC-induced deaths even with curative resection (Huang et al., 2019; Sakamoto et al., 2019). Therefore, we focused on the lncRNAs implicated in the metastasis and recurrence of HCC. Via analyzing The Cancer Genome Atlas (TCGA) Liver Hepatocellular Carcinoma (LIHC) dataset, we identified a novel lncRNA LINC01134, which is associated with poor survival of HCC patients. The expression and function of LINC01134 in human cancers have not been investigated. In this study, we further detected the expression and clinical significances of LINC01134 in HCC. Using *in vitro* and *in vivo* gain- and loss-of-function experiments, we found that LINC01134 promotes HCC cell migration and invasion and HCC liver metastasis and lung metastasis. Mechanistically, we found that LINC01134 directly binds the promoter of *AKT1S1* and activates *AKT1S1* expression. Via activating AKT1S1, LINC01134 further activates NF-κB signaling. Our findings unveiled that LINC01134 may be a potential therapeutic target against HCC metastasis.

## Materials and Methods

### Tissue Specimens

Eighty-four pairs of HCC tissues and paired adjacent noncancerous liver tissues and 20 portal vein tumor thrombus (PVTT) tissues were obtained from HCC patients who received surgery at Tongji Hospital (Wuhan, China) with written informed consent. None of the patients received chemotherapy and/or radiotherapy before surgery. The clinical parameters of these 84 HCC patients were obtained retrospectively from pathology reports and listed in Table 1. All tissue specimens were confirmed by pathological examination. Tissue specimens were acquired during surgery and immediately snap-frozen in liquid nitrogen and stored at −80°C until use. The Ethics Committee of Tongji Hospital (Wuhan, China) reviewed and approved this study.

**TABLE 1:** Relationship between the LINC01134 levels and clinicopathological features of 84 HCC patients.

**Variable**	***n***	**LINC01134 level**		***p* value**
		**Low**	**High**	
Age				0.498
≥55	31	14	17	
<55	53	28	25	
Gender				0.533
Male	72	37	35	
Female	12	5	7	
HBs antigen				0.693
Positive	77	38	39	
Negative	7	4	3	
Liver cirrhosis				0.595
With	66	32	34	
Without	18	10	8	
Serum AFP concentration				0.147
≥20 μg/L	60	27	33	
<20 μg/L	24	15	9	
Tumor size				0.382
>5 cm	44	20	24	
≤5 cm	40	22	18	
Microvascular invasion				0.047
Present	22	7	15	
Absent	62	35	27	
Macrovascular invasion				0.024
Present	11	2	9	
Absent	73	40	33	
Encapsulation				0.079
Complete	38	23	15	
No	46	19	27	

**p*-Value was calculated by Pearson’s chi-square tests. Microvascular invasion is defined as microscopic tumor invasion in smaller intrahepatic vessels identified on pathologic analysis. Macrovascular invasion is defined as invasion of tumor into a major vessel that can be identified during macroscopic examination or radiological imaging.*

### Cell Culture and Treatment

Human HCC cell lines SK-HEP-1, HCCLM3, and Huh7 were acquired from the Cell Bank of Type Culture Collection (Chinese Academy of Sciences, Shanghai, China). The cells were cultured in Dulbecco’s modified Eagle’s medium (DMEM, Invitrogen, Carlsbad, CA, United States) supplemented with 10% fetal bovine serum (FBS, Invitrogen) and incubated at 37°C in a humidified incubator with 5% CO_2_. Where indicated, the HCC cells were treated with 20 nM rapamycin (Selleck, Houston, TX, United States) or 5 μM JSH-23 (Selleck) for 48 h.

### RNA Extraction and Quantitative RT-PCR

Total RNA was extracted from indicated tissues and cells using the TRIzol reagent (Cat# 15596-026, Invitrogen) strictly following the manufacturer’s instruction. RNA concentration and quality were detected by the NanoDrop spectrophotometry (Thermo Fisher Scientific) with the A260/A280 ratios being allowed between 1.8 and 2.2. Next, cDNA was synthesized using the PrimeScript^TM^ II First-Strand cDNA Synthesis Kit (Cat# 6210A, Takara, Dalian, China) strictly following the manufacturer’s instruction. Quantitative RT-PCR (qRT-PCR) was carried out using the TB Green^®^ Premix Ex Taq^TM^ (Tli RNaseH Plus), ROX plus (Cat# RR42LR, Takara) on an ABI StepOnePlus real-time PCR system (Applied Biosystems, Irvine, CA, United States). The amplification conditions were as follows: 95°C for 30 s, followed by 40 cycles of 95°C for 5 s, 60°C for 30 s, and 72°C for 30 s. Amplification efficiencies are allowed to be between 95 and 105%. The primers used were as follows: for LINC01134 (LINC01134-202), 5′-CATATTTGAAAGGGGCAGAC-3′ (forward) and 5′-GGCAACATTAGCCAAACCTA-3′ (reverse); for LINC01134-201, 5′-CCGTTTCTCCGGTGCTATC-3′ (forward) and 5′-TTCTCCGCTGTGTCCTTCAT-3′ (reverse); for LINC01134-203, 5′-GCCATGTTTGAGCGAGGAA-3′ (forward) and 5′-AAGGAGGAGGATGGGAGTC-3′ (reverse); for LINC01134-204, 5′-TCACAGGCCTTGGGCCAGT-3′ (forward) and 5′-GCGTGGGAAACATAGCGGC-3′ (reverse); for AKT1S1, 5′-GAGGGCTCTTTGTGATGGATG-3′ (forward) and 5′-TGCTGTGTGGGTAGGGCTGA-3′ (reverse); for E-cadherin, 5′-GCCCCATCAGGCCTCCGTTT-3′ (forward) and 5′-ACCTTGCCTTCTTTGTCTTTGTTGGA-3′ (reverse); for vimentin, 5′-CCTGAACCTGAGGGAAACTAA-3′ (forward) and 5′-GCAGAAAGGCACTTGAAAGC-3′ (reverse); for NEAT1, 5′-GTCTTTCCATCCACTCACGTCT-3′ (forward) and 5′-GGACAACTAGATGCCGAGGTAG-3′ (reverse); for MALAT1, 5′-GGATCCTAGACCAGCATGCC-3′ (forward) and 5′-AAAGGTTACCATAAGTAAGTTCCAGAAAA-3′ (reverse); and for GAPDH, 5′-GGTCTCCTCTGACTTCAACA-3′ (forward) and 5′-GTGAGGGTCTCTCTCTTCCT-3′ (reverse). GAPDH was selected as endogenous control. The relative expression of RNAs was calculated using the −2^ΔΔ*Ct*^ method.

### Subcellular Fractionation

Subcellular fractionation was carried out as described before (Gagnon et al., 2014). The RNA in different subcellular components was extracted and detected by qRT-PCR as described above.

### Vector Construction and Transfection

LINC01134 full-length sequences were generated by PCR with the primers 5′-GGAATTCACACTGGAGCAGGAAGTC-3′ (forward) and 5′-GCTCTAGACCATATGAGAATGAAGGTTTT-3′ (reverse). Next, the LINC01134 sequences were cloned into the *Eco*RI and *Xba*I sites of pcDNA^TM^3.1(+) vector (Invitrogen) to generate the LINC01134 overexpression vector. LINC01134 full-length sequences with the deletion of 464 to 753 nt were synthesized by GenScript (Nanjing, China) and cloned into the pcDNA^TM^3.1(+) vector to generate the mutated LINC01134 overexpression vector. Two independent cDNA oligonucleotides repressing LINC01134 (shLINC-1 and shLINC-2) and one cDNA oligonucleotide repressing AKT1S1 (shAKT1S1) were synthesized by GenePharma (Shanghai, China) and cloned into the GenePharma SuperSilencing^TM^ shRNA expression vector pGPU6/Hygro. The sequences of shLINC-1 were 5′-CACCGGACAGGTTTGAGCTAGAAACTTCAAGAGAGTTT CTAGCTCAAACCTGTCCTTTTTTG-3′ (forward) and 5′-GATCCAAAAAAGGACAGGTTTGAGCTAGAAACTCTCTT GAAGTTTCTAGCTCAAACCTGTCC-3′ (reverse). The sequences of shLINC-2 were 5′-CACCGCGCATCCACTCAT TCACTCATTCAAGAGATGAGTGAATGAGTGGATGCGCTT TTTTG-3′ (forward) and 5′-GATCCAAAAAAGCGCATCCA CTCATTCACTCATCTCTTGAATGAGTGAATGAGTGGATG CGC-3′ (reverse). The sequences of shAKT1S1 were 5′-CACCGGAAACAGGACCTCCTCTAGATTCAAGAGATCTAG AGGAGGTCCTGTTTCCTTTTTTG-3′ (forward) and 5′-GA TCCAAAAAAGGAAACAGGACCTCCTCTAGATCTCTTGAA TCTAGAGGAGGTCCTGTTTCC-3′ (reverse). The sequences of negative control shRNA (shNC) were 5′-CAC CGTTCTCCGAACGTGTCACGTCAAGAGATTACGTGACAC GTTCGGAGAATTTTTTG-3′ (forward) and 5′-GATCCAAA AAATTCTCCGAACGTGTCACGTAATCTCTTGACGTGACA CGTTCGGAGAAC-3′ (reverse). *AKT1S1* promoter sequences were generated by PCR with the primers 5′-GGGGTACCCTCCAGCATCACCTCTTCC-3′ (forward) and 5′-CCCAAGCTTGCCTACTCACCCACTTCGT-3′ (reverse) and then cloned into the *Kpn*I and *Hin*dIII sites of pGL3-basic Luciferase Reporter Vector (Promega, Madison, WI, United States) to generate the *AKT1S1* promoter reporter pGL3-AKT1S1. Transfection of vectors was undertaken using Lipofectamine 3000 (Invitrogen) according to the manufacturer’s instruction.

### Stable Cell Line Construction

To construct LINC01134-stably-overexpressed HCC cells, LINC01134 overexpression vector and control pcDNA^TM^3.1(+) vector were transfected into SK-HEP-1 and HCCLM3 cells. Forty-eight hours after transfection, the cells were treated with 800 μg/ml neomycin for 4 weeks to select LINC01134-overexpressed SK-HEP-1 and HCCLM3 cells. To construct LINC01134-stably-silenced HCC cells, shLINC-1, shLINC-2, and shNC were transfected into HCCLM3 and Huh7 cells. Seventy-two hours after transfection, the cells were treated with 800 μg/ml hygromycin for 4 weeks to select LINC01134-silenced HCCLM3 and Huh7 cells. To construct LINC01134-overexpressed and concurrently AKT1S1-silenced HCC cells, shAKT1S1 and shNC were transfected into LINC01134-stably-overexpressed SK-HEP-1 and HCCLM3 cells. Seventy-two hours after transfection, the cells were treated with 800 μg/ml neomycin and 800 μg/ml hygromycin for 4 weeks to select LINC01134-overexpressed and concurrently AKT1S1-silenced SK-HEP-1 and HCCLM3 cells. To construct luciferase-labeled cells, indicated HCC cells were infected with luciferase-expressing lentivirus (Ubi-MCS-firefly_Luciferase-IRES-Puromycin) (Cat# LVCON101, GeneChem, Shanghai, China) and selected with 2 μg/ml puromycin for 4 weeks to construct luciferase stably labeled HCC cells.

### Transwell Migration and Invasion Assays

Cell migration and invasion abilities were assessed by Transwell migration and invasion assays, respectively. Transwell chambers (Corning, NY, United States) coated with Matrigel were used for Transwell invasion assay. The same Transwell chambers coated without Matrigel were used for Transwell migration assay. A total of 50,000 indicated HCC cells resuspended in FBS-free medium were plated into the upper chambers pre-coated with or without Matrigel. Complete medium was added to the lower chambers. After 48 h of incubation, the cells remaining on the upper chambers were removed. The migratory and invasive cells were fixed with paraformaldehyde and stained with crystal violet. At least five random fields were photographed under a photomicroscope (Zeiss, Oberkochen, Germany) to count the cell numbers.

### Cell Viability Assay

Cell viability was measured using the Cell Counting Kit-8 (CCK-8) assay. Plated into 96-well plate per well were 3,000 indicated HCC cells. The cells were concurrently treated with 20 nM rapamycin (Cat# S1039, Selleck). At the indicated time, cell viability was measured using the CCK-8 (Cat# CK04, Dojindo Laboratories) following the manufacturer’s protocol.

### Mouse Xenograft Models

Six-week-old male athymic BALB/c nude mice were used in this study, and the Ethics Committee of Tongji Hospital (Wuhan, China) reviewed and approved the mouse xenograft assays. For liver metastasis experiments, 3 × 10^6^ indicated HCC cells were intrasplenically injected into nude mice. The mice were allowed to grow for another 4 weeks. Then, the mice were sacrificed, and the liver were resected, embedded in paraffin, and used to carry out routine hematoxylin–eosin (HE) staining. The number and size of liver metastatic nodules were assessed under a photomicroscope. For lung metastasis experiments, 2 × 10^6^ luciferase-labeled indicated HCC cells were injected into tail veins of nude mice. The mice were allowed to grow for another 4 weeks. Lung metastases were monitored using the IVIS@ Lumina II system (Caliper Life Sciences, Hopkinton, MA, United States) 10 min after intraperitoneal injection of 4.0 mg of luciferin in 50 μl of saline.

### Western Blot

Total protein was isolated from indicated HCC cells using RIPA lysis buffer (Beyotime, Shanghai, China) supplemented with protease inhibitor PMSF (Beyotime). After quantitation of protein concentration with Enhanced BCA Protein Assay Kit (Beyotime), an equal amount of protein was separated by sodium dodecyl sulfate-polyacrylamide gel electrophoresis, followed by being transferred to a polyvinylidene fluoride membrane (Beyotime). After blocking, the membrane was incubated with primary antibodies against AKT1S1 (ab151719, 1:1,000, Abcam, Cambridge, MA, United States), phospho-AKT1S1 (ab226994, 1:1,000, Abcam), p65 (#8242, 1;1,000, Cell Signaling Technology), histone H3 (ab1791, 1:1,000, Abcam), or GAPDH (ab8245, 1:10,000, Abcam). After three washes, the membrane was further incubated with goat anti-rabbit IgG H&L (IRDye^®^ 800CW) preadsorbed (ab216773, 1:10,000, Abcam) or goat anti-mouse IgG H&L (IRDye^®^ 680RD) preadsorbed (ab216776, 1:10,000, Abcam). Lastly, the membrane was detected on the Odyssey infrared scanner (Li-Cor, Lincoln, NE, United States).

### Chromatin Isolation by RNA Purification

Chromatin isolation by RNA purification (ChIRP) was carried out using the Magna ChIRP^TM^ RNA Interactome Kit (Millipore, Bedford, MA, United States) following the manufacturer’s instruction. The sequences of the antisense DNA probes targeting LINC01134 were as follows: 1, 5′-gaaacatagcggctacaggg-3′; 2, 5′-aagcgccgttcacgaacatg-3′; 3, 5′-attttactttcaggcctttc-3′; 4, 5′-ttcaagtggtttctagctca-3′; 5, 5′-ataggtcttggctggttctc-3′; 6, 5′-gcaaacagcgaggtcaacac-3′; 7, 5′-cgagggaggacactgacatg-3′; 8, 5′-cgcagaatggcaggaatcaa-3′; 9, 5′-gaatgttcaggagagagggc-3′; 10, 5′-aaggtaggttctgggacatg-3′; and 11, 5′-gagtgggggtggtcactaac-3′. The enriched DNA was detected by qRT-PCR with the primers 5′-AGAGCGAGCCAGGACTTG-3′ (forward) and 5′-GGTATCTTATTGTGGTTTTGG-3′ (reverse) for the *AKT1S1* promoter and 5′-TGTCACTACCGCAGAGCCT-3′ (forward) and 5′-GAGGACTTTGGGAACGACTG-3′ (reverse) for the *GAPDH* promoter.

### Dual Luciferase Reporter Assays

To assess the effects of LINC01134 on *AKT1S1* promoter activity, the *AKT1S1* promoter reporter pGL3-AKT1S1 was co-transfected with pRL-TK and LINC01134 overexpression or silencing vectors into HCCLM3 cells. pRL-TK encodes renilla luciferase and was used as an endogenous reference. Forty-eight hours after transfection, the firefly luciferase and renilla luciferase activities were measured using the Dual-Luciferase Reporter Assay System (Promega). To assess the effects of LINC01134 on NF-κB transcriptional activity, firefly luciferase reporter containing NF-κB binding sites (pNFκB-luc) (Beyotime) was co-transfected with pRL-TK and LINC01134 overexpression or silencing vectors into HCCLM3 cells. Forty-eight hours after transfection, the firefly luciferase and renilla luciferase activities were measured using the Dual-Luciferase Reporter Assay System (Promega).

### p65-DNA Binding Activity Assay

Forty-eight hours after transfection of LINC01134 overexpression or silencing vectors into HCCLM3 cells, the nuclear extracts from transfected cells were used to carry out p65-DNA binding activity with an NF-κB p65 Transcription Factor Assay Kit (ab133112, Abcam) following the manufacturer’s instruction.

### Statistical Analysis

GraphPad Prism v6.0 (GraphPad Software, La Jolla, CA, United States) was used to undergo all statistical analyses. For comparison, Wilcoxon matched-pairs signed-rank test, log-rank test, Pearson’s chi-square test, unpaired two-sided Student’s *t*-test, one-way ANOVA followed by Dunnett’s multiple comparisons test, one-way ANOVA followed by Tukey’s multiple comparisons test, Mann–Whitney test, Kruskal–Wallis test followed by Dunn’s multiple comparisons test, and Spearman correlation analysis were performed as indicated. *P* < 0.05 was regarded as statistically significant.

## Results

### LINC01134 Is Highly Expressed and Correlated With Poor Survival of HCC Patients

**FIGURE 1:**
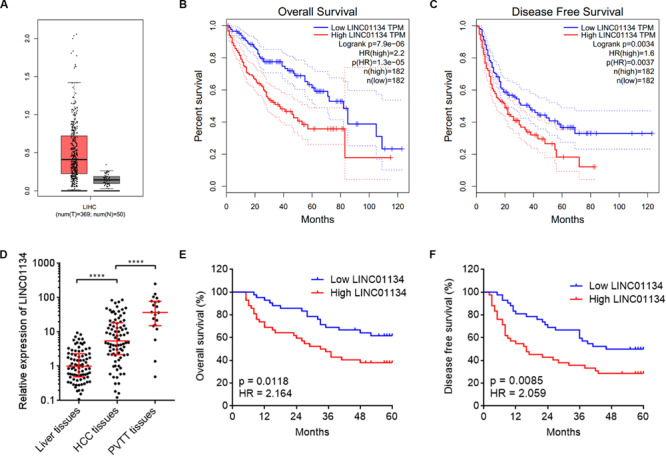
LINC01134 is highly expressed and correlated with poor prognosis of HCC patients. **(A)** The expression of LINC01134 in HCC tissues (*n* = 369) versus normal liver tissues (*n* = 50) from TCGA LIHC dataset. **(B)** Kaplan–Meier survival analysis of the correlation between LINC01134 expression levels and overall survival of HCC patients from TCGA LIHC dataset. **(C)** Kaplan–Meier survival analysis of the correlation between LINC01134 expression levels and disease-free survival of HCC patients from TCGA LIHC dataset. **(D)** The expression of LINC01134 in 84 pairs of HCC tissues and paired adjacent noncancerous liver tissues and 20 PVTT tissues was detected by qRT-PCR. ****p < 0.0001. The comparison between liver tissues and HCC tissues was calculated by Wilcoxon matched-pairs signed-rank test. The comparison between HCC tissues and PVTT tissues was calculated by Mann–Whitney test. **(E)** Kaplan–Meier survival analysis of the correlation between LINC01134 expression level and overall survival of these 84 HCC patients. *p* = 0.0118, HR = 2.164 by log-rank test. **(F)** Kaplan–Meier survival analysis of the correlation between LINC01134 expression level and disease-free survival of these 84 HCC patients. *p* = 0.0085, HR = 2.059 by log-rank test.

To identify the genes correlated with survival of HCC patients, we analyzed TCGA LIHC dataset using the online tool GEPIA.^1^ The top 100 genes most significantly correlated with overall survival of HCC patients are shown in Supplementary Table 1. Although most of these genes are protein-coding genes, we noted a gene (ID: ENSG00000236423.5) which encodes an lncRNA LINC01134. Reanalyzing TCGA LIHC dataset, we found that LINC01134 is significantly highly expressed in HCC tissues (*n* = 369) compared with normal liver tissues (*n* = 50) (Figure 1A). Reanalyzing TCGA LIHC dataset with available survival data, we found that high levels of LINC01134 indicate shorter overall survival and disease-free survival times (Figures 1B,C). Searching Ensembl,^2^ we found that the LINC01134 has five exons with four isoforms (Supplementary Figure 1A). Quantitative RT-PCR with isoform-specific primers indicated that LINC01134-202 is the most abundant isoform in HCCLM3 cells and HCC tissues (Supplementary Figures 1B,C). Therefore, we focused on the most abundant isoform LINC01134-202. Three *in silico* tools, CPC, CPAT, and PhyloCSF, all predicted that LINC01134 as a ncRNA (Supplementary Figures 1D–F). To further explore the clinical significance of LINC01134, we collected 84 pairs of HCC tissues and paired adjacent noncancerous liver tissues. Quantitative RT-PCR results revealed that LINC01134 is significantly highly expressed in HCC tissues compared with noncancerous liver tissues (Figure 1D). In addition, we collected 20 PVTT tissues, which are intrahepatic metastases of HCC. qRT-PCR results revealed that LINC01134 is further highly expressed in PVTT tissues compared with HCC tissues (Figure 1D). Analyzing the correlation between LINC01134 expression levels and the clinical parameters revealed that LINC01134-highly-expressed HCC tissues showed more microvascular invasion and macrovascular invasion compared to LINC01134-lowly-expressed HCC tissues (Table 1). LINC01134-highly-expressed HCC patients had significantly shorter overall survival and disease-free survival compared to LINC01134-lowly-expressed HCC patients (Figures 1E,F).

### LINC01134 Promotes Migration and Invasion of HCC Cells

**FIGURE 2:**
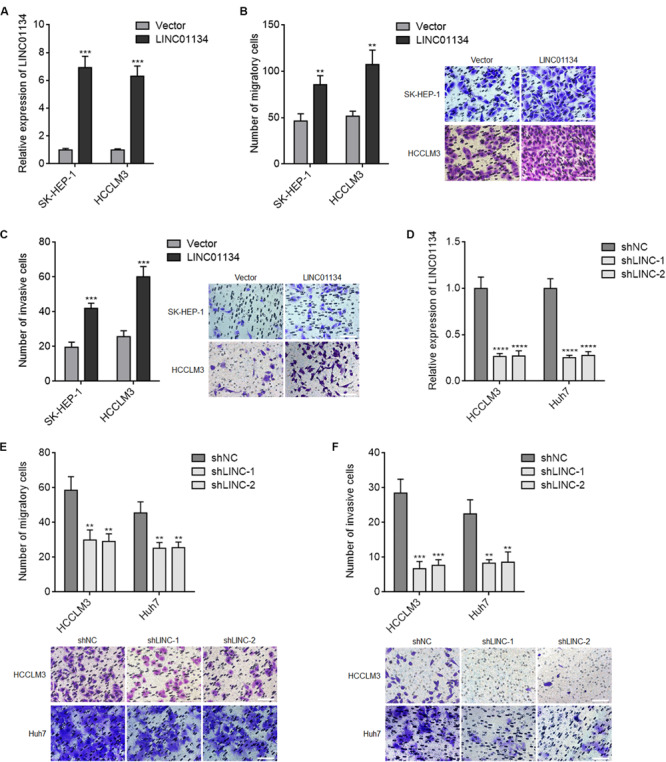
LINC01134 promotes migration and invasion of HCC cells. **(A)** The expression of LINC01134 in LINC01134-stably-overexpressed and control SK-HEP-1 and HCCLM3 cells was detected by qRT-PCR. **(B)** Transwell migration assays were conducted to assess the migration ability of LINC01134-stably-overexpressed and control SK-HEP-1 and HCCLM3 cells. Representative images of migratory cells were shown. Scale bar = 100 μm. **(C)** Transwell invasion assays were conducted to assess the invasion ability of LINC01134-stably-overexpressed and control SK-HEP-1 and HCCLM3 cells. Representative images of invasive cells were shown. Scale bar = 100 μm. **(D)** The expression of LINC01134 in LINC01134-stably-silenced and control HCCLM3 and Huh7 cells was detected by qRT-PCR. **(E)** Transwell migration assays were conducted to assess the migration ability of LINC01134-stably-silenced and control HCCLM3 and Huh7 cells. Representative images of migratory cells were shown. Scale bar = 100 μm. **(F)** Transwell invasion assays were conducted to assess the invasion ability of LINC01134-stably-silenced and control HCCLM3 and Huh7 cells. Representative images of invasive cells were shown. Scale bar = 100 μm. Results are shown as the mean ± SD of three independent experiments. **p < 0.01, ***p < 0.001, ****p < 0.0001 by unpaired two-sided Student’s *t*-test **(A–C)** or one-way ANOVA followed by Dunnett’s multiple comparisons test **(D–F)**.

Due to LINC01134 being positively correlated with microvascular invasion, macrovascular invasion, and poor survival of HCC patients, we next investigated the roles of LINC01134 in HCC cell migration and invasion. LINC01134-stably-overexpressed HCC cells were constructed via stable transfection of LINC01134 overexpression plasmids into SK-HEP-1 and HCCLM3 cells. The overexpression efficiencies were confirmed by qRT-PCR (Figure 2A). Transwell migration assays showed that LINC01134-overexpressed SK-HEP-1 and HCCLM3 cells have stronger migratory ability compared to control SK-HEP-1 and HCCLM3 cells, respectively (Figure 2B). Transwell invasion assays showed that LINC01134-overexpressed SK-HEP-1 and HCCLM3 cells have stronger invasive ability compared to control SK-HEP-1 and HCCLM3 cells, respectively (Figure 2C). Furthermore, LINC01134-stably-silenced HCC cells were constructed via stable transfection of two independent LINC01134-specific shRNAs into HCCLM3 and Huh7 cells. The knockdown efficiencies were confirmed by qRT-PCR (Figure 2D). Transwell migration assays showed that LINC01134-silenced HCCLM3 and Huh7 cells have less migratory cell number compared to control HCCLM3 and Huh7 cells, respectively (Figure 2E). Transwell invasion assays showed that LINC01134-silenced HCCLM3 and Huh7 cells have less invasive cell number compared to control HCCLM3 and Huh7 cells, respectively (Figure 2F). Due to relationships between EMT, cell migration, and invasion, we next investigated whether LINC01134 regulates EMT in HCC cells. Epithelial and mesenchymal markers E-cadherin and vimentin, respectively, were assayed in LINC01134-overexpressing SK-HEP-1 and LINC01134-silenced HCCLM3 cells, by qRT-PCR. The results showed that neither LINC01134 overexpression nor LINC01134 silencing modulates EMT marker expression (Supplementary Figures 2A,B), which suggested that LINC01134 did not modulate EMT in HCC cells. Collectively, these data demonstrated that LINC01134 promotes HCC cell migration and invasion.

### LINC01134 Promotes HCC Liver Metastasis and Lung Metastasis *in vivo*

**FIGURE 3:**
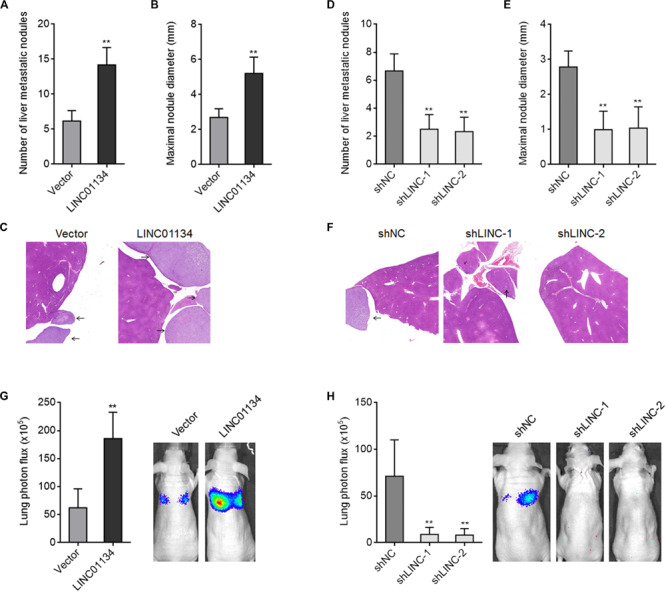
LINC01134 promotes HCC liver metastasis and lung metastasis *in vivo*. **(A–C)** LINC01134-stably-overexpressed and control HCCLM3 cells were intrasplenically injected into nude mice. At the 28th day after injection, the number **(A)** and size **(B)** of liver metastatic nodules were detected by HE staining **(C)**. **(D–F)** LINC01134-stably-silenced and control HCCLM3 cells were intrasplenically injected into nude mice. At the 28th day after injection, the number **(D)** and size **(E)** of liver metastatic nodules were detected by HE staining **(F)**. **(G)** Luciferase-labeled LINC01134-stably-overexpressed and control HCCLM3 cells were injected into the tail veins of nude mice. At the 28th day after injection, lung metastasis was assessed via detecting luciferase signal intensities. **(H)** Luciferase-labeled LINC01134-stably-silenced and control HCCLM3 cells were injected into the tail veins of nude mice. At the 28th day after injection, lung metastasis was assessed via detecting luciferase signal intensities. Results are shown as the mean ± SD of *n* = 6 mice in each group. **p < 0.01 by Mann–Whitney test **(A–C,G)** or Kruskal–Wallis test followed by Dunn’s multiple comparisons test **(D–F,H)**.

To investigate the effects of LINC01134 in HCC liver metastasis *in vivo*, LINC01134-stably-overexpressed and control HCCLM3 cells were intrasplenically injected into nude mice. At the 28th day after injection, the liver metastatic nodules were detected by HE staining. As shown in Figures 3A–C, LINC01134-overexpressed HCCLM3 cells form more and larger liver metastatic nodules compared to control cells. LINC01134-stably-silenced and control HCCLM3 cells were also intrasplenically injected into nude mice. At the 28th day after injection, the liver metastatic nodules were detected by HE staining. As shown in Figures 3D–F, LINC01134-silenced HCCLM3 cells form less and smaller liver metastatic nodules compared to control cells. Next, the effects of LINC01134 on HCC lung metastasis were explored. Luciferase-labeled LINC01134-stably-overexpressed and control HCCLM3 cells were injected into the tail veins of nude mice. At the 28th day after injection, LINC01134-overexpressed HCCLM3 cells form more lung metastasis compared to control cells (Figure 3G). Luciferase-labeled LINC01134-stably-silenced and control HCCLM3 cells were also injected into the tail veins of nude mice. At the 28th day after injection, LINC01134-silenced HCCLM3 cells form less lung metastasis compared to control cells (Figure 3H). Therefore, these data suggested that LINC01134 promotes HCC liver metastasis and lung metastasis *in vivo*.

### The Expression of AKT1S1 Is Positively Correlated With That of LINC01134 in HCC Tissues

**FIGURE 4:**
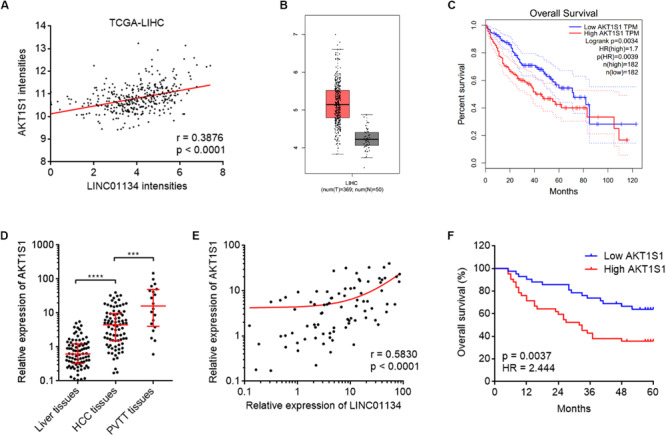
The positive correlation between AKT1S1 and LINC01134 expressions in HCC tissues. **(A)** The correlation between AKT1S1 and LINC01134 expression intensities from TCGA LIHC dataset. *r* = 0.3876, *p* < 0.0001 by Spearman correlation analysis. **(B)** The expression of AKT1S1 in HCC tissues (*n* = 369) versus normal liver tissues (*n* = 50) from TCGA LIHC dataset. **(C)** Kaplan–Meier survival analysis of the correlation between AKT1S1 expression levels and overall survival of HCC patients from TCGA LIHC dataset. **(D)** The expression of AKT1S1 in 84 pairs of HCC tissues and paired adjacent noncancerous liver tissues and 20 PVTT tissues was detected by qRT-PCR. ****p < 0.0001. The comparison between liver tissues and HCC tissues was calculated by Wilcoxon matched-pairs signed-rank test. The comparison between HCC tissues and PVTT tissues was calculated by Mann–Whitney test. **(E)** The correlation between LINC01134 expression level and AKT1S1 expression level in these 84 HCC tissues. *r* = 0.583, *p* < 0.0001 by Spearman correlation analysis. **(F)** Kaplan–Meier analyses of the correlation between AKT1S1 expression level and overall survival of these 84 HCC patients. *p* = 0.0037, HR = 2.444 by log-rank test.

To explore the potential molecular mechanisms mediating the roles of LINC01134 in HCC, we searched the genes whose expressions are correlated with those of LINC01134 in TCGA LIHC dataset using the online tool R2^3^ (Supplementary Table 2). Among the genes most significantly correlated with LINC01134, we noted AKT1S1, which has been reported to be a critical oncogene in several cancers, including HCC (Malla et al., 2015; Lv et al., 2017; Qi et al., 2020). The correlation between LINC01134 and AKT1S1 expression intensities based on TCGA LIHC dataset is shown in Figure 4A. Analyzing TCGA LIHC dataset, we found that in line with LINC01134, AKT1S1 is also significantly highly expressed in HCC tissues (*n* = 369) compared with normal liver tissues (*n* = 50) (Figure 4B). Reanalyzing TCGA LIHC dataset with available survival data, we found that in line with LINC01134, high levels of AKT1S1 indicate shorter overall survival time (Figure 4C). In our cohort containing 84 pairs of HCC tissues and paired adjacent noncancerous liver tissues and additional 20 PVTT tissues, we further confirmed that AKT1S1 is highly expressed in HCC tissues compared with noncancerous liver tissues and further highly expressed in PVTT tissues compared with HCC tissues (Figure 4D). The expression of AKT1S1 is also significantly positively correlated with that of LINC01134 in these 84 HCC tissues (Figure 4E). AKT1S1-highly-expressed HCC patients had significantly shorter overall survival compared to AKT1S1-lowly-expressed HCC patients (Figure 4F). The correlation between the expressions of LINC01134, AKT1S1, and EMT markers in TCGA LIHC dataset was further analyzed using R2. The results showed that the expressions of LINC01134 and AKT1S1 are not consistently correlated with EMT markers in TCGA LIHC dataset (Supplementary Figures 2C–F), which supported that LINC01134 and AKT1S1 did not modulate EMT in HCC.

### LINC01134 Activates *AKT1S1* Transcription via Directly Binding the Promoter of *AKT1S1*

**FIGURE 5:**
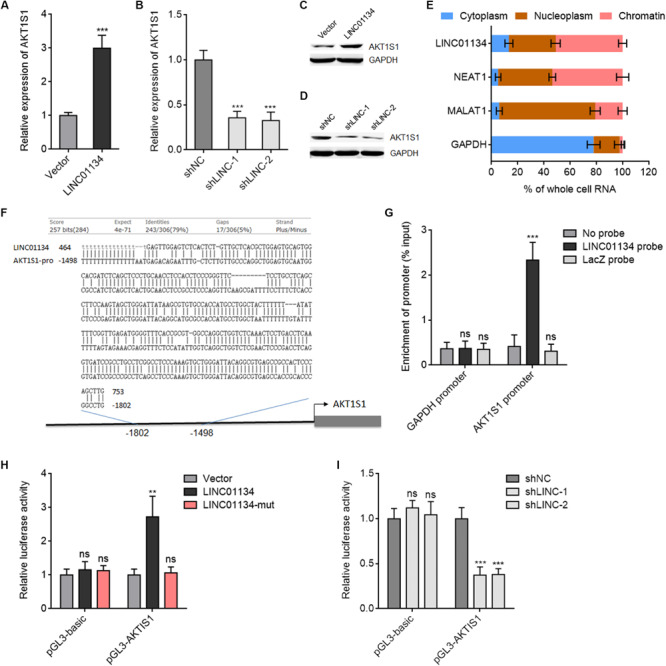
LINC01134 directly binds the *AKT1S1* promoter and activates *AKT1S1* expression. **(A)** AKT1S1 mRNA levels in LINC01134-stably-overexpressed and control HCCLM3 cells were detected by qRT-PCR. **(B)** AKT1S1 mRNA levels in LINC01134-stably-silenced and control HCCLM3 cells were detected by qRT-PCR. **(C)** AKT1S1 protein levels in LINC01134-stably-overexpressed and control HCCLM3 cells were detected by western blot. **(D)** AKT1S1 protein levels in LINC01134-stably-silenced and control HCCLM3 cells were detected by western blot. **(E)** Subcellular localization of LINC01134 and control transcripts was analyzed by qRT-PCR in biochemically fractionated HCCLM3 cells. NEAT1 was used as a chromatin-associated lncRNA control. MALAT1 was used as a nucleoplasm-localized lncRNA control. GAPDH mRNA was used as a cytoplasmic control. **(F)** The highly adversely complementary region between LINC01134 (464–753 nt) and *AKT1S1* promoter (−1,802 to −1,498 bp). **(G)** ChIRP assays with LINC01134 capture probes were conducted in HCCLM3 cells. The enrichment of the *AKT1S1* promoter and *GAPDH* promoter was detected by qRT-PCR. **(H)** After transient co-transfection of wild-type or complementary region mutated LINC01134 overexpression plasmids with firefly luciferase reporter containing the *AKT1S1* promoter and pRL-TK (encoding renilla luciferase) into HCCLM3 cells, dual luciferase reporter assays were conducted to assess AKT1S1 promoter activity. Results are shown as the relative ratio of firefly luciferase activity to renilla luciferase activity. **(I)** After transient co-transfection of LINC01134-specific shRNAs with firefly luciferase reporter containing the *AKT1S1* promoter and pRL-TK into HCCLM3 cells, dual luciferase reporter assays were conducted to assess AKT1S1 promoter activity. Results are shown as the relative ratio of firefly luciferase activity to renilla luciferase activity. Results are shown as the mean ± SD of three independent experiments. **p < 0.01, ***p < 0.001, ns, not significant, by unpaired two-sided Student’s *t*-test **(A)** or one-way ANOVA followed by Dunnett’s multiple comparisons test **(B,G–I)**.

Due to the significantly positive correlation between LINC01134 and AKT1S1 expressions in HCC tissues, we next investigated whether LINC01134 regulates AKT1S1 in HCC. AKT1S1 mRNA and protein levels in LINC01134-stably-overexpressed and LINC01134-silenced HCCLM3 cells were detected by qRT-PCR and western blot. As shown in Figures 5A–D, LINC01134-overexpressed HCCLM3 cells have significantly higher mRNA and protein expression of AKT1S1 compared to control cells, while LINC01134-silenced HCCLM3 cells have significantly lower mRNA and protein expression of AKT1S1 compared to control cells. These data suggested that LINC01134 activates AKT1S1 expression. To investigate the potential mechanisms underlying the modulation of AKT1S1 by LINC01134, we first assessed the subcellular localization of LINC01134 via biochemical fractionation. The results revealed that LINC01134 is mainly localized in the chromatin (Figure 5E), suggesting a potential role of LINC01134 in regulating gene transcription. Intriguingly, we identified a highly adversely complementary region between LINC01134 (464–753 nt) and the promoter of *AKT1S1* (−1,802 to −1,498 bp) (Figure 5F). To explore whether LINC01134 directly binds the *AKT1S1* promoter via this complementary region, ChIRP assays were conducted with biotinylated LINC01134 capture probes. As shown in Figure 5G, the *AKT1S1* promoter is specifically enriched in the LINC01134 probe group, but not in the control probe group (LacZ probe). To investigate whether the binding of LINC01134 to the *AKT1S1* promoter modulates *AKT1S1* transcription, the promoter of *AKT1S1* containing the LINC01134 binding site was cloned into the firefly luciferase reporter. Dual luciferase reporter assays showed that concurrent overexpression of LINC01134 significantly increased the luciferase activity of the *AKT1S1* promoter, which was abrogated by the mutation of the complementary sequences on LINC01134 (Figure 5H). Conversely, LINC01134 knockdown significantly decreased the luciferase activity of the *AKT1S1* promoter (Figure 5I). Collectively, these data suggested that LINC01134 activates *AKT1S1* transcription via directly binding the *AKT1S1* promoter.

### AKT1S1 Silencing Reverses the Roles of LINC01134 in Promoting HCC Migration, Invasion, and Metastasis

**FIGURE 6:**
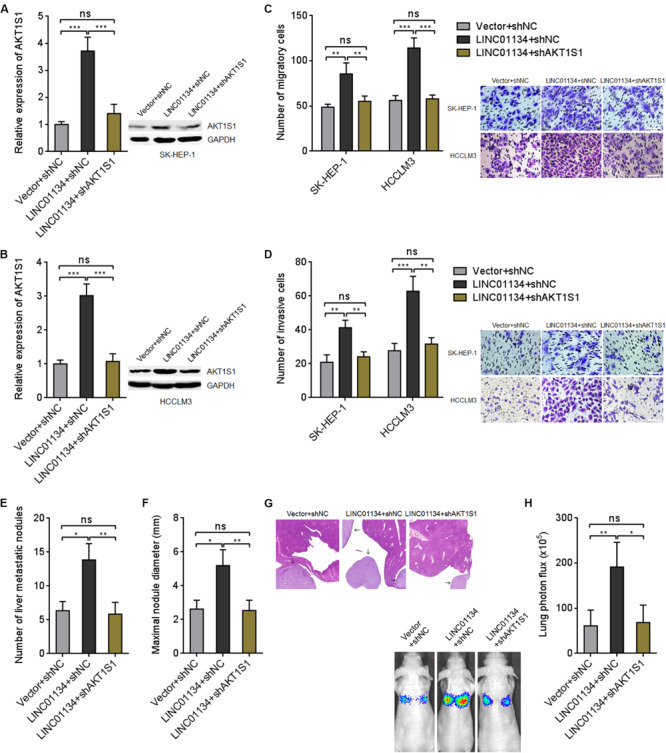
Inhibition of AKT1S1 reverses the roles of LINC01134 in HCC. **(A)** AKT1S1 mRNA and protein levels in LINC01134-stably-overexpressed and concurrently AKT1S1-stably-silenced SK-HEP-1 cells were detected by qRT-PCR and western blot. **(B)** AKT1S1 mRNA and protein levels in LINC01134-stably-overexpressed and concurrently AKT1S1-stably-silenced HCCLM3 cells were detected by qRT-PCR and western blot. **(C)** Transwell migration assays were conducted to assess the migration ability of LINC01134-stably-overexpressed and concurrently AKT1S1-stably-silenced SK-HEP-1 and HCCLM3 cells. Representative images of migratory cells were shown. Scale bar = 100 μm. **(D)** Transwell invasion assays were conducted to assess invasion ability of LINC01134-stably-overexpressed and concurrently AKT1S1-stably-silenced SK-HEP-1 and HCCLM3 cells. Representative images of invasive cells were shown. Scale bar = 100 μm. **(E–G)** LINC01134-stably-overexpressed and concurrently AKT1S1-stably-silenced HCCLM3 cells were intrasplenically injected into nude mice. At the 28th day after injection, the number **(E)** and size **(F)** of liver metastatic nodules were detected by HE staining **(G)**. **(H)** Luciferase-labeled LINC01134-stably-overexpressed and concurrently AKT1S1-stably-silenced HCCLM3 cells were injected into the tail veins of nude mice. At the 28th day after injection, lung metastasis was assessed via detecting luciferase signal intensities. Results are shown as the mean ± SD of three independent experiments **(A–D)** or *n* = 6 mice in each group **(E–H)**. *p < 0.05, **p < 0.01, ***p < 0.001, ns, not significant, by one-way ANOVA followed by Tukey’s multiple comparisons test **(A–D)** or Kruskal–Wallis test followed by Dunn’s multiple comparisons test **(E,F,H)**.

To explore whether the activation of AKT1S1 mediates the roles of LINC01134 in promoting HCC migration, invasion, and metastasis, AKT1S1 was stably silenced in LINC01134-stably-overexpressed SK-HEP-1 and HCCLM3 cells via stable transfection of AKT1S1-specific shRNAs (Figures 6A,B). Transwell migration assays revealed that the pro-migratory roles of LINC01134 are abolished by AKT1S1 silencing (Figure 6C). Transwell invasion assays revealed that the pro-invasive roles of LINC01134 are also abolished by AKT1S1 silencing (Figure 6D). LINC01134-stably-overexpressed and AKT1S1-stably-silenced HCCLM3 cells were intrasplenically injected into nude mice. At the 28th day after injection, the liver metastatic nodules were detected. As shown in Figures 6E–G, the increased number and size of liver metastatic nodules caused by LINC01134 overexpression are abolished by AKT1S1 silencing. Luciferase-labeled LINC01134-stably-overexpressed and AKT1S1-stably-silenced HCCLM3 cells were also injected into the tail veins of nude mice. At the 28th day after injection, the increased lung metastasis caused by LINC01134 overexpression is abolished by AKT1S1 silencing (Figure 6H). Rapamycin, a known mammalian target of rapamycin (mTOR) pathway inhibitor, is reported to inhibit AKT1S1 (Hu et al., 2015). We then treated LINC01134-stably-overexpressed SK-HEP-1 and HCCLM3 cells with rapamycin to repress AKT1S1 (Supplementary Figures 3A,B). Rapamycin treatment suppressed cell viability of HCC cells (Supplementary Figures 3C,D). Transwell migration assays showed that the pro-migratory roles of LINC01134 are abolished by rapamycin (Supplementary Figure 3E). Transwell invasion assays showed that the pro-invasive roles of LINC01134 are also abolished by rapamycin (Supplementary Figure 3F). These data supported the concept that LINC01134 promotes HCC migration and invasion via activation of AKT1S1.

### LINC01134 Enhances NF-κB Transcriptional Activity in HCC

**FIGURE 7:**
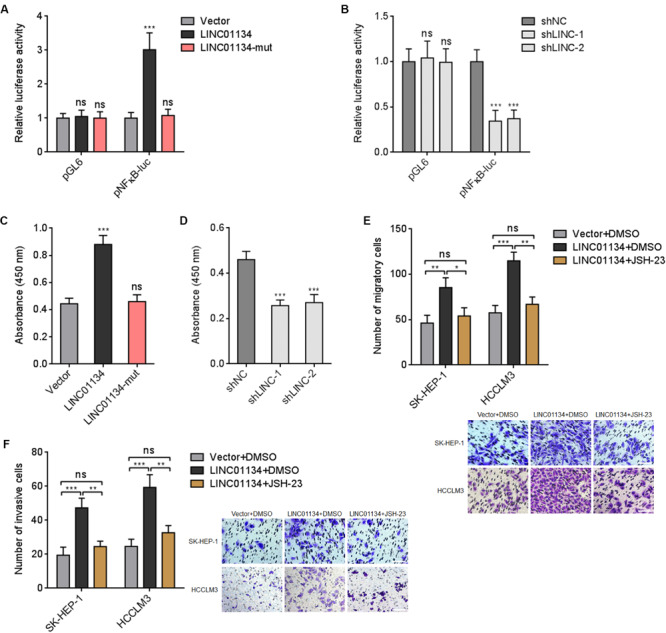
LINC01134 enhances NF-κB transcriptional activity. **(A)** After transient co-transfection of wild-type or complementary region mutated LINC01134 overexpression plasmids with firefly luciferase reporter containing NF-κB binding sites (pNFκB-luc) and pRL-TK into HCCLM3 cells, dual luciferase reporter assays were conducted to assess NF-κB transcriptional activity. Results are shown as the relative ratio of firefly luciferase activity to renilla luciferase activity. **(B)** After transient co-transfection of LINC01134-specific shRNAs with firefly luciferase reporter containing NF-κB binding sites (pNFκB-luc) and pRL-TK into HCCLM3 cells, dual luciferase reporter assays were conducted to assess NF-κB transcriptional activity. Results are shown as the relative ratio of firefly luciferase activity to renilla luciferase activity. **(C)** After transient transfection of wild-type or complementary region mutated LINC01134 overexpression plasmids into HCCLM3 cells, the p65-DNA binding activity was detected using an NF-κB p65 Transcription Factor Assay Kit. **(D)** After transient transfection of LINC01134-specific shRNAs into HCCLM3 cells, the p65-DNA binding activity was detected using an NF-κB p65 Transcription Factor Assay Kit. **(E)** LINC01134-stably-overexpressed and control SK-HEP-1 and HCCLM3 cells were treated with 5 μM JSH-23. Then, the migration ability of treated cells was assessed by Transwell migration assays. Representative images of migratory cells were shown. Scale bar = 100 μm. **(F)** LINC01134-stably-overexpressed and control SK-HEP-1 and HCCLM3 cells were treated with 5 μM JSH-23. Then, the invasion ability of treated cells was assessed by Transwell invasion assays. Representative images of invasive cells were shown. Scale bar = 100 μm. Results are shown as the mean ± SD of three independent experiments. **p < 0.01, ***p < 0.001, ns, not significant, by one-way ANOVA followed by Dunnett’s multiple comparisons test **(A–D)** or one-way ANOVA followed by Tukey’s multiple comparisons test **(E,F)**.

Previous report has shown that AKT1S1 enhances NF-κB transcriptional activity via associating with p65 (Zhu et al., 2017). Thus, we further investigated whether LINC01134 modulates NF-κB signaling via the activation of AKT1S1. Dual luciferase reporter assays showed that overexpression of LINC01134 significantly increases NF-κB transcriptional activity, which is abrogated by the mutation of the complementary sequences on LINC01134 (Figure 7A). Conversely, knockdown of LINC01134 significantly reduces NF-κB transcriptional activity (Figure 7B). The effects of LINC01134 on p65-DNA binding activity were further investigated. As shown in Figure 7C, overexpression of LINC01134 significantly increases p65-DNA binding activity, which is abrogated by the mutation of the complementary sequences on LINC01134. Conversely, knockdown of LINC01134 reduces p65-DNA binding activity (Figure 7D). To further investigate whether the roles of LINC01134 in promoting HCC cell migration and invasion are dependent on the regulation of AKT1S1-NF-κB signaling, LINC01134-stably-overexpressed SK-HEP-1 and HCCLM3 cells were treated with NF-κB signaling inhibitor JSH-23, which repressed p65 nuclear translocation (Supplementary Figure 4). Transwell migration assays showed that treatment with JSH-23 abrogated the pro-migratory roles of LINC01134 (Figure 7E). Transwell invasion assays showed that treatment with JSH-23 abrogated the pro-invasive roles of LINC01134 (Figure 7F). Thus, these data suggested that the activation of AKT1S1-NF-κB transcriptional activity at least partially mediates the roles of LINC01134 in promoting HCC cell migration and invasion.

## Discussion

Recent advances in high-throughput sequencings have discovered more and more lncRNAs in diverse types of cancers (Iyer et al., 2015; Berger et al., 2018). Growing studies suggest the important clinical significances and roles of lncRNAs in many cancers, including HCC (Yuan et al., 2014). In this study, we provide another evidence for the implication of lncRNA in HCC.

In this study, we identified a relative novel lncRNA LINC01134. Although this lncRNA has been included in the National Center for Biotechnology Information (NCBI), the expression and roles of LINC01134 in human diseases have not been reported. To our knowledge, we first found that LINC01134 is significantly highly expressed in HCC tissues compared to noncancerous liver tissues. Increased expression of LINC01134 is positively correlated with microvascular invasion and macrovascular invasion but not correlated with age, gender, hepatitis B surface antigen (HBs antigen), liver cirrhosis, alpha-fetoprotein (AFP), tumor size, and encapsulation. Furthermore, high expression of LINC01134 is correlated with poor disease-free survival and overall survival. Our findings identified LINC01134 as an indicator of HCC recurrence and prognosis. The best cutoff values of LINC01134 could be obtained from larger and multicenter cohorts to predict patients’ survival. LINC01134 is 1,965 nt in length, and the gene encoding LINC01134 is located at chromosome 1p36.32. Analyzing TCGA dataset, we found that LINC01134 is also highly expressed in stomach cancer, prostate cancer, lung cancer, esophageal cancer, and bladder cancer and correlated with poor survival of adrenocortical carcinoma, kidney cancer, lung cancer, and glioma patients. These data suggest that LINC01134 may be a cancer-correlated lncRNA. The expressions and clinical significances of LINC01134 in other cancers need further detection.

Gain- and loss-of-function experiments showed that enhanced expression of LINC01134 promotes HCC cell migration and invasion *in vitro* and HCC liver metastasis and lung metastasis *in vivo*. Silencing of LINC01134 represses HCC cell migration and invasion *in vitro* and HCC liver metastasis and lung metastasis *in vivo*. Our findings identified LINC01134 as a lncRNA regulating HCC metastasis and suggested LINC01134 as a potential therapeutic target for HCC metastasis. Whether LINC01134 regulates the metastasis of other cancers need further investigation.

Different subcellular distributions of lncRNAs influence the different mechanisms of action of lncRNAs. For cytoplasmic lncRNAs, they could directly bind microRNAs and relieve the repressive roles of microRNAs on their genuine targets (Yuan et al., 2014). Furthermore, the cytoplasmic lncRNAs could bind proteins and change the posttranslational modification and/or stability of the interacted proteins (Wang et al., 2014). For nuclear lncRNAs, they often directly bind epigenetic modification enzymes, such as EZH2, and epigenetically regulate the transcription of target genes (Sun et al., 2016). Nuclear lncRNAs may also directly bind DNA, change the architecture of chromatin, and further modulate the transcription of target genes (Ding et al., 2016). In this study, we identified LINC01134 as a chromatin-binding lncRNA and found that LINC01134 directly binds the promoter of *AKT1S1*. AKT1S1, also known as PRAS40, is a substrate of Akt and a component of the mTOR complex 1 (Volkers and Sussman, 2013). The oncogenic roles of AKT1S1 have been reported in colon cancer, liver cancer, lung cancer, prostate cancer, breast cancer, and so on via regulating PI3K/Akt, mTOR, and/or NF-κB signaling pathways (Malla et al., 2015; Lv et al., 2017). In this study, we found a significantly positive correlation between LINC01134 and AKT1S1 expressions in HCC tissues. In line with LINC01134, AKT1S1 is also highly expressed in HCC and correlated with poor survival of HCC patients. Via binding the promoter of *AKT1S1*, LINC01134 activates *AKT1S1* expression and further activates NF-κB signaling. Functional rescue experiments revealed that silencing AKT1S1 or blocking NF-κB signaling reversed the roles of LINC01134. Thus, our findings identified the activation of AKT1S1-NF-κB signaling as the critical mediators of the roles of LINC01134 in HCC. AKT1S1 could be phosphorylated by Akt and mTOR (Lv et al., 2017). Phosphorylated AKT1S1 was reported to promote HCC metastasis (Hu et al., 2015). In this study, we found that LINC01134 upregulates the total AKT1S1 protein level and also phospho-AKT1S1 level. The further downstream mechanisms after AKT1S1 phosphorylation in HCC metastasis need further investigation. Nevertheless, our findings identified AKT1S1 as a critical mediator of the roles of LINC01134 in promoting HCC metastasis. The effects of LINC01134–*AKT1S1* promoter binding on the chromatin architecture of the *AKT1S1* promoter need further investigation to elucidate *AKT1S1* activation mechanisms. NF-κB signaling pathways are involved in various cancers, including HCC. NF-κB signaling modulates many biological behaviors of cancer cells, including proliferation, apoptosis, migration, invasion, and metastasis (Xu et al., 2013; Wang et al., 2015; Duan et al., 2018; Rodrigues et al., 2018). In this study, we mainly focused on their roles in HCC metastasis. Through regulating NF-κB signaling, the effects of LINC01134 and AKT1S1 on HCC cell proliferation and apoptosis need further investigation. During our revision, another group reported that LINC01134 promotes EMT and metastasis of HCC through the LINC01134/miR-324-5p/IGF3BP1/YY1 axis (Rong et al., 2020). We both reported the pro-metastatic roles of LINC01134 in HCC. But we identified different molecular mechanisms mediating the roles of LINC01134 in HCC, which reflect the diversity and complexity of lncRNAs’ roles and mechanisms in different cellular contexts. Nonetheless, we both identified LINC01134 as a potential therapeutic target for HCC.

## Conclusion

**FIGURE 8:**
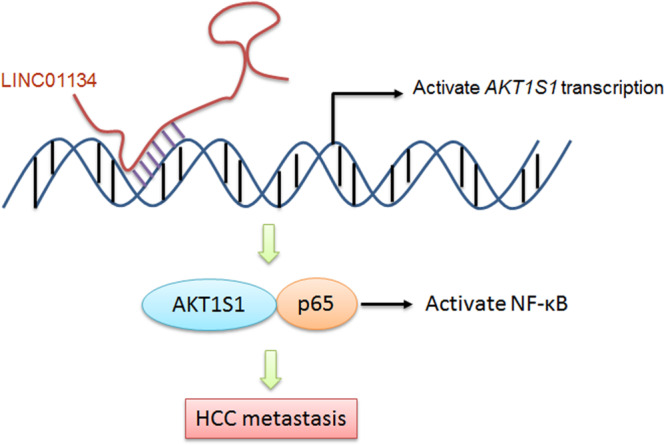
A schematic model of LINC01134 in promoting HCC metastasis via activating AKT1S1-NF-κB signaling.

Taken together, in this study, we identified a novel lncRNA, LINC01134, which is highly expressed in HCC tissues compared to noncancerous liver tissues. The expression of LINC01134 is positively correlated with microvascular invasion and macrovascular invasion. Furthermore, LINC01134 indicates recurrence and poor overall survival of HCC patients. LINC01134 was identified as a chromatin-binding lncRNA and directly binds the promoter of *AKT1S1* and subsequently activates *AKT1S1* transcription. Via activating AKT1S1, LINC01134 further activates NF-κB signaling. Via activating AKT1S1-NF-κB signaling, LINC01134 promotes HCC cell migration and invasion *in vitro* and metastasis *in vivo* (Figure 8). Our findings suggested LINC01134 as a potential prognostic biomarker and therapeutic target for HCC.

## Data Availability Statement

The raw data supporting the conclusions of this article will be made available by the authors, without undue reservation, to any qualified researcher.

## Ethics Statement

The studies involving human participants were reviewed and approved by the Ethics Committee of Tongji Hospital (Wuhan, China). The patients/participants provided their written informed consent to participate in this study.

## Author Contributions

LZ and KC designed this study. CW, YC, and LZ carried out the experiments. LZ, KC, and CW analyzed the data. LZ and CW wrote the manuscript. All authors have read and approved the final manuscript.

## Conflict of Interest

The authors declare that the research was conducted in the absence of any commercial or financial relationships that could be construed as a potential conflict of interest.

## Footnotes

1 http://gepia.cancer-pku.cn/detail.php

2 http://asia.ensembl.org/index.html

3 https://hgserver1.amc.nl/cgi-bin/r2/main.cgi
