# Supplementary material for: Long Noncoding RNA LINC01134 Promotes Hepatocellular Carcinoma Metastasis via Activating AKT1S1 and NF-κB Signaling
Source: Front Cell Dev Biol. 2020 Jun 12;8:429. doi: 10.3389/fcell.2020.00429 (PMC7325970; doi:10.3389/fcell.2020.00429)
Supplement: Supplementary file 1 [file Image_1.pdf]

Supplementary Material

1 Supplementary Figures 1–4

1.1 Supplementary Figure 1

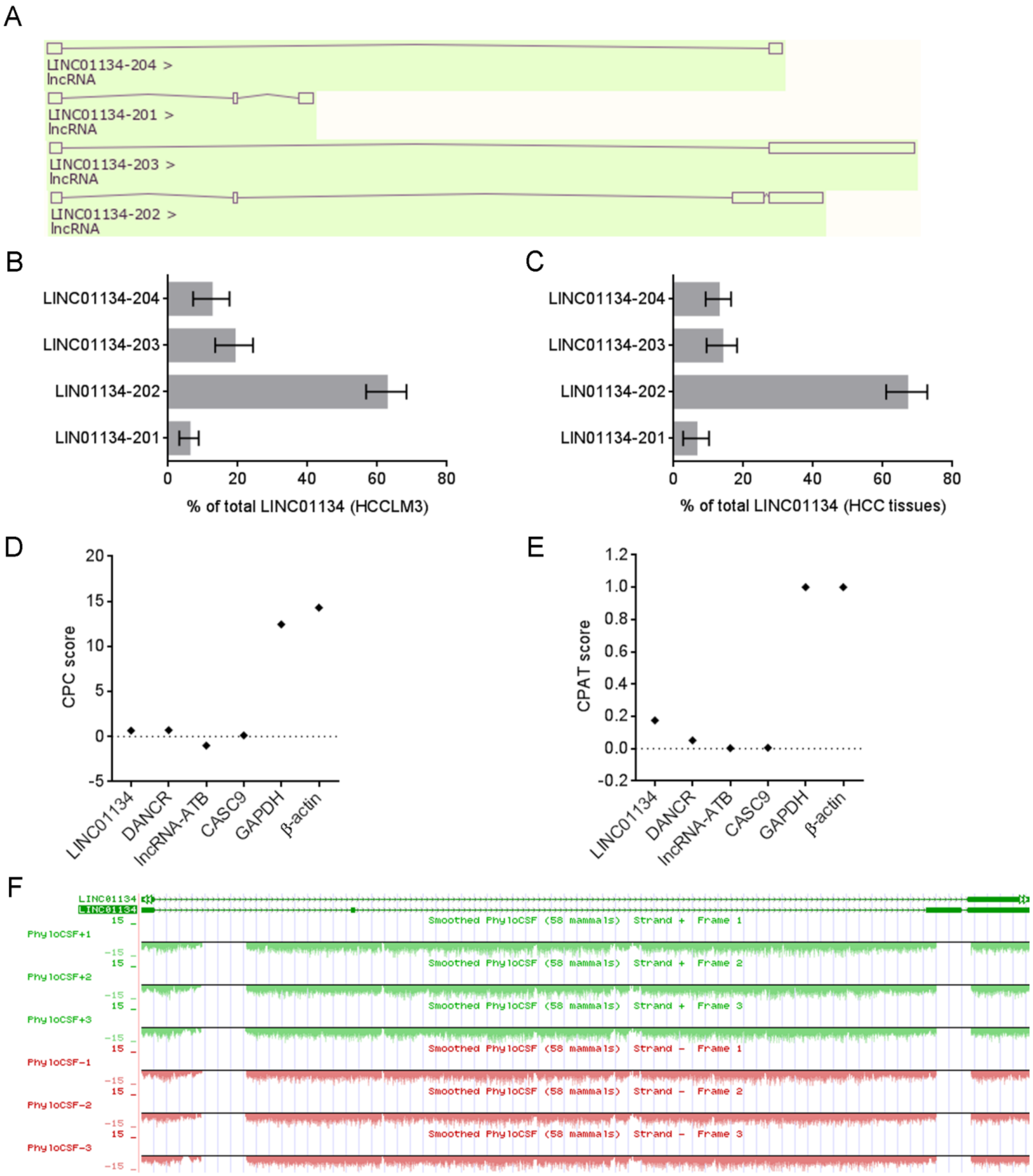

Supplementary Figure 1. The characteristics of LINC01134. **(A)** The exon maps for the four isoforms. **(B)** The expression levels of LINC01134 isoforms in HCCLM3 cells were detected by qRT-PCR with isoform-specific primers. Results are shown as the mean ± SD of three independent experiments. **(C)** The expression levels of LINC01134 isoforms in HCC tissues were detected by qRT-PCR with isoform-specific primers. Results are shown as the mean ± SD of three independent experiments. **(D)** Protein coding capacity of LINC01134 and control transcripts was determined by CPC *in silico* tools. **(E)** Protein coding capacity of LINC01134 and control transcripts was determined by CPAT *in silico* tools. **(F)** UCSC genome browser view of PhyloCSF tracks for LINC01134.

1.2 Supplementary Figure 2

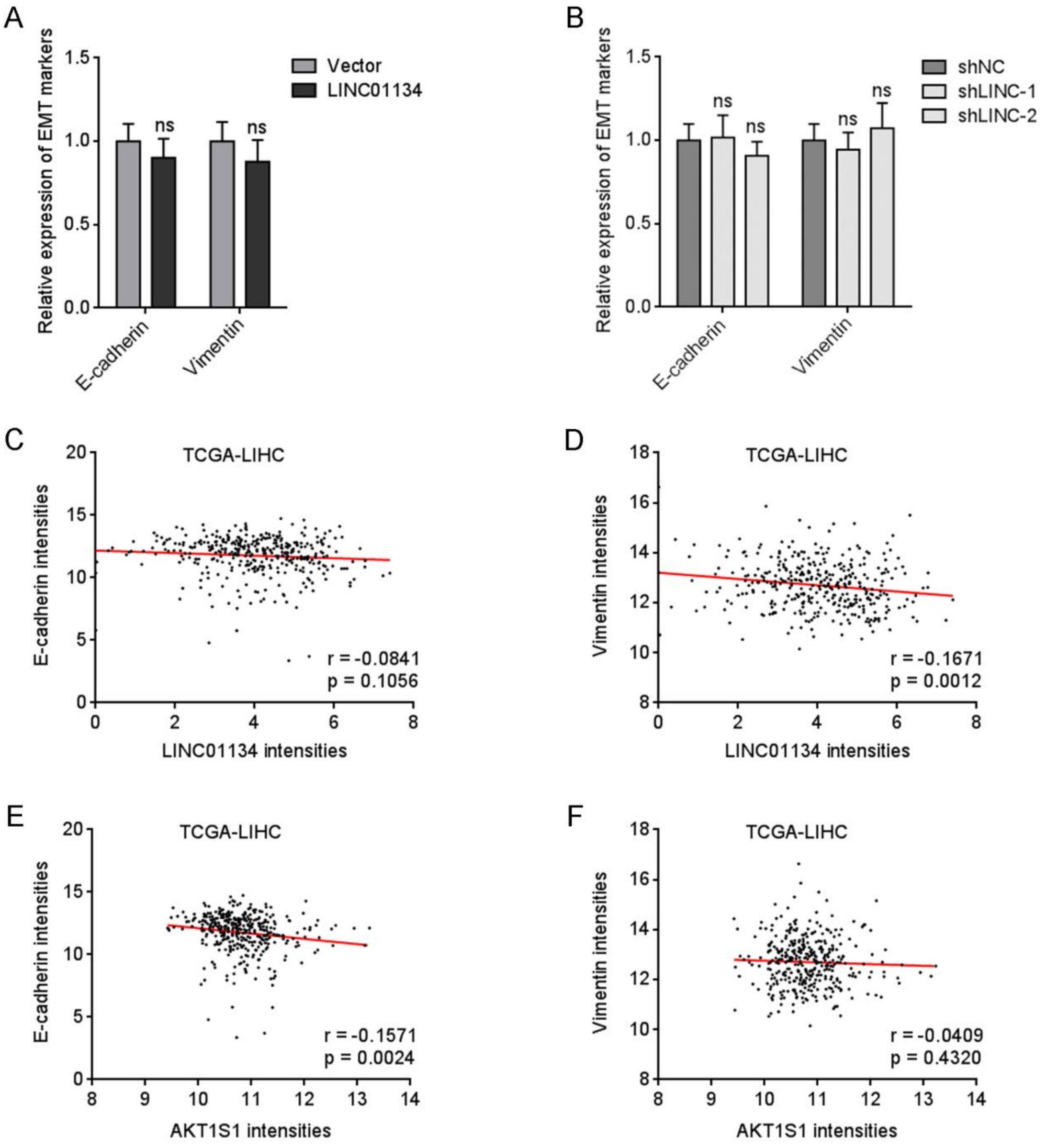

Supplementary Figure 2. LINC01134 and AKT1S1 do not modulate EMT. (**A**) The expression of E-cadherin and Vimentin in LINC01134 stably overexpressed and control SK-HEP-1 cells was detected by qRT-PCR. ns, not significant, by unpaired two-sided Student's *t*-test. **(B)** The expression of E-cadherin and Vimentin in LINC01134 stably silenced and control HCCLM3 cells was detected by qRT-PCR. ns, not significant, by one-way ANOVA followed by Dunnett's multiple comparisons test. (**C**) The correlation between LINC01134 and E-cadherin expression intensities from TCGA LIHC dataset. r = -0.0841, p = 0.1056 by Spearman correlation analysis. (**D**) The correlation between LINC01134 and Vimentin expression intensities from TCGA LIHC dataset. r = -0.1671, p = 0.0012 by Spearman correlation analysis. (**E**) The correlation between AKT1S1 and E-cadherin expression intensities from TCGA LIHC dataset. r = -0.1571, p = 0.0024 by Spearman correlation analysis. (**F**) The correlation between AKT1S1 and Vimentin expression intensities from TCGA LIHC dataset. r = -0.0409, p = 0.4320 by Spearman correlation analysis.

1.3 Supplementary Figure 3

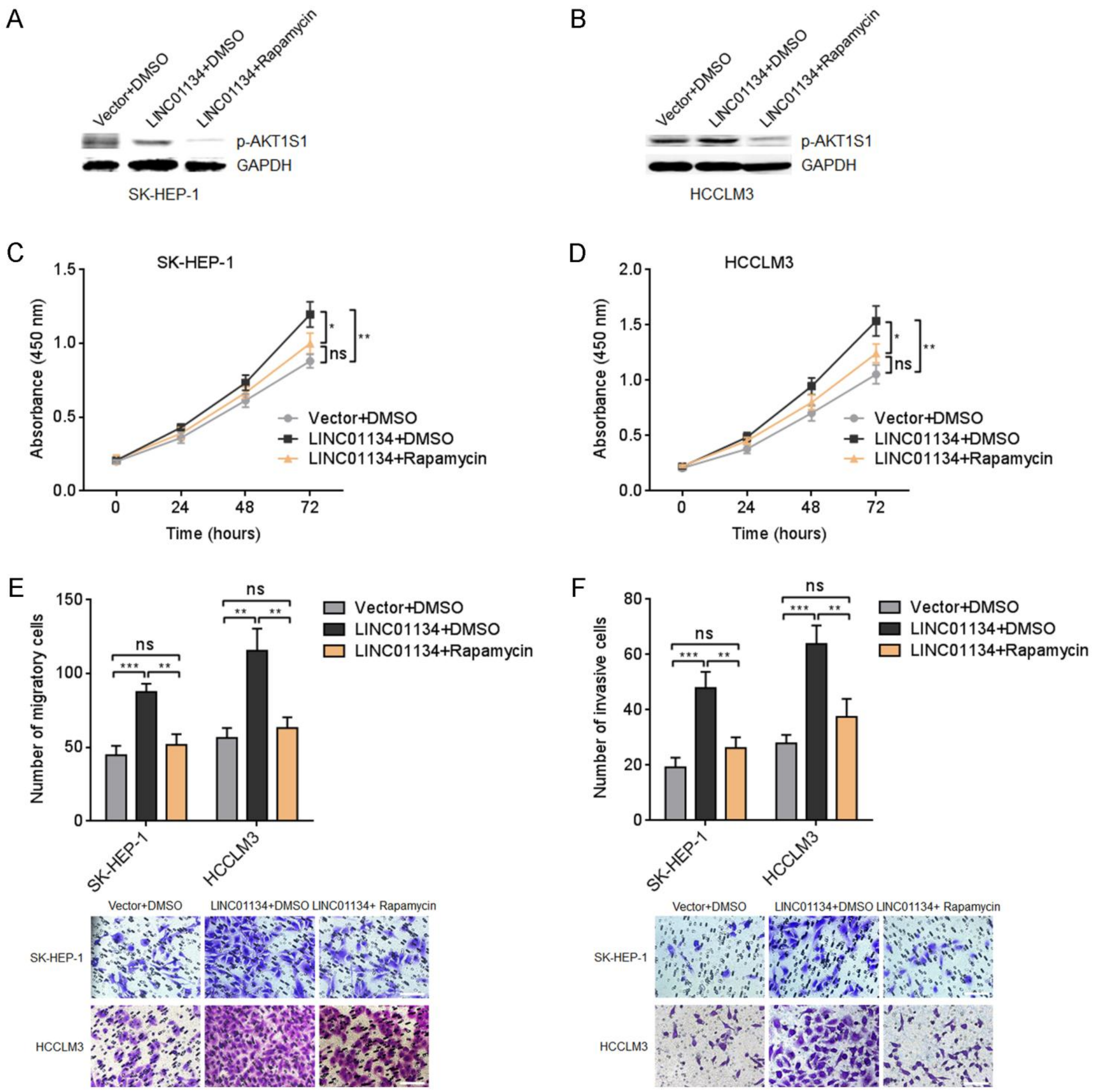

Supplementary Figure 3. Inhibition of AKT1S1 by rapamycin reverses the roles of LINC01134 in HCC. **(A)** AKT1S1 phosphorylation levels in LINC01134 stably overexpressed and control SK-HEP-1 cells pretreated with 20 nM rapamycin for 48 hours were detected by western blot. **(B)** AKT1S1 phosphorylation levels in LINC01134 stably overexpressed and control HCCLM3 cells pretreated with 20 nM rapamycin for 48 hours were detected by western blot. **(C)** LINC01134 stably overexpressed and control SK-HEP-1 cells were treated with 20 nM rapamycin. Concurrently, cell proliferation was assessed by CCK-8 assays. **(D)** LINC01134 stably overexpressed and control

HCCLM3 cells were treated with 20 nM rapamycin. Concurrently, cell proliferation was assessed by CCK-8 assays. **(E)** LINC01134 stably overexpressed and control SK-HEP-1 and HCCLM3 cells were treated with 20 nM rapamycin. Concurrently, migration ability of treated cells was assessed by transwell migration assays. Representative images of migratory cells were shown. Scale bar = 100 μm. **(F)** LINC01134 stably overexpressed and control SK-HEP-1 and HCCLM3 cells were treated with 20 nM rapamycin. Concurrently, invasion ability of treated cells was assessed by transwell invasion assays. Representative images of invasive cells were shown. Scale bar = 100 μm. Results are shown as the mean ± SD of three independent experiments. $^{*}p < 0.05$, $^{**}p < 0.01$, $^{***}p < 0.001$, ns, not significant, by one-way ANOVA followed by Tukey's multiple comparisons test.

1.4 Supplementary Figure 4

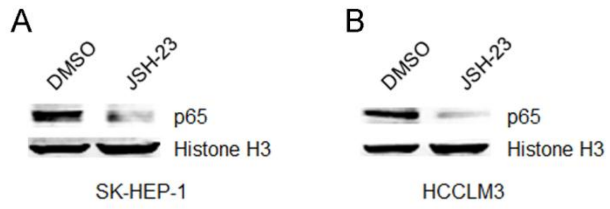

Supplementary Figure 4. JSH-23 represses p65 nuclear translocation. (**A**) Nuclear p65 levels in LINC01134 overexpressed SK-HEP-1 cells pretreated with 5 μM JSH-23 or DMSO for 48 hours was detected by western blot. (**B**) Nuclear p65 levels in LINC01134 overexpressed HCCLM3 cells pretreated with 5 μM JSH-23 or DMSO for 48 hours was detected by western blot.
